# Condensations from Late Editorials in the Medical Press

**Published:** 1881-05

**Authors:** 


### Condensations from Late Editorials in the Medical Press.

THE NATIONAL ASSOCIATION FOR THE PROTECTION OF THE INSANE held a meeting in Boston recently which was notable in many ways. * * One of the topics of discussion, opened by a letter from Dr. Joseph F. Parrish, was that of the etiology and prevention of insanity. Dr. Parrish referred to the fact that insanity arose so largely among a wearied and restless class in whom bodily and mental vigor were sadly disproportionate. He believed that there ought to be more institutions for the treatment of persons who are only on the border-line of insanity, in the "crazy circle" of Crichton-Brown. Dr. H. B. Wilbur, referring to the same subject, expressed his belief that insanity depended on predisposing causes—hereditary, social, and individual—rather than exciting causes. While our civilization increased it, education and study did not, if applied in a proper way. Dr. Nathan Allen said that in his experience ill-health caused more insanity than any other agency. Dr. Allen and Dr. Kenniston, in a subsequent speech, asserted that intemperance was a great cause of insanity, and that any diminution in the former would equally affect the latter. Dr. W. W. Gooding feared that we must accept brain disease as one of the penalties of a higher development, hinting a possible future arrest of a growth of mind aspiring to be god-like.—*Medical Record.*

They seem to overlook the fact that mental disease is often a result of uterine diseases in the female; and that, as Dr. William Hammond asserts, a great many cases of insanity are easily traced to superstition, to disappointed love, and other excesses of passions.

THE ABOLITION OF THE OFFICE OF CORONER.—In attempting to reform the present system it is necessary to take into consideration the abolition of the coroner's jury, the division of the office of coroner into two distinct functions, and the creation of a new system of appointment whereby fitness for the duties will be guaranteed. These are, on their face, reasonable propositions, and are capable of easy fulfillment by the enactment of a suitable law. The passage of such a law could be urged, not only on the ground of general utility, but on the score of economy. It has been proved, time and again, that the verdict of the coroner's jury has no possible bearing upon the subsequent judicial proceedings; in a word, is useless as a judicial measure, gives no security to the accused, is burdensome upon the citizen, and expensive to the State. The coroner, as an officer, is of no importance, qualified neither for medical nor judicial examinations; he is practically a direct incumbrance to the course of justice. All the valuable part of his services is performed by his deputy, who is the medical examiner. The combination of the duties of two offices into one could be made without any possible loss to the State as to actual service, while the salary of the coroner would be saved. Under the Massachusetts law, the saving to the State in criminal proceedings has been one-third with the advantage of the services of skilled experts, and the smooth, prompt working of a perfect judicial process.—*Medical Record.*

---

THE HARVARD MEDICAL SCHOOL, AND REFORMS IN MEDICAL EDUCATION.—The annual report of Harvard University for the year 1881 is extremely interesting to those who care to know what progress is being made in elevating our methods of medical education. The medical department of this university is in a prosperous condition, and has great reason to congratulate itself upon the good work that it has done. The whole number of students in attendance during the three terms of the past year averaged two hundred and fifty-one. An appreciation of the advantages of spending the time of study at the college is shown by the fact that eighty-six per cent. of the graduates in the above year had been in attendance during six collegiate terms. The difference between this condition of things and that in most other medical schools, where two terms is the maximum, is very striking. The plan of introducing preliminary examinations has had an extremely favorable effect upon the *personnel* of the college. Nearly one-half the students have literary or scientific degrees."—*Medical Record.*

The proportion of students who came up for the examination and were rejected was nearly one-third.

---

THE DECLINE OF THE BIRTH RATE.—Some German Scientists, after a careful investigation into the causes of poverty, misery and crime, have come to the conclusion that an excessively high birth-rate is a frequent cause. This has also been the conclusion of every poor man who has had to rear a large family since the days of Adam. It is wonderful how philosophers revel in highly sounding words facts, which the most ignorant never doubted. Whatever the opposition which the doctrine of evolution may encounter, mankind practically knows that the first and necessary quality of an adult man is to be self-sustaining, and he must be more than that if he marries, for he must now sustain a wife and children ; and the number of these he will endeavor to limit according to his powers of sustenance, if not influenced by other motives than those of preservation. John Bascom, in a late work on the Science of Duty, says that only the poor and the improvident think that they have a right to multiply indefinitely. By looking at the subject in another light, I believe, one must come to the conclusion that the strongest plea for public prostitution rests on that same fact : the inability of one to provide for a numerous offspring, or even for a wife. Although it is also a fact that sexual life absorbs a large share of the thoughts and actions of men.

As there is no end to discussions of this character, especially now that we discard empirical views, and refer to the uniform course of nature, what are the practical results to be gathered from well-known facts? 1. Marriage should be delayed until both contractants are able to provide for themselves and a few children; this is a great conflict with the natural feelings of mankind, for it does away with the most fecund years of the female and induces marriage after the twenty-fifth or thirtieth year. 2. Right or wrong, the prevention of conception has been practically tested in this country as well as in France, and gives results which seem very satisfactory to the parents, but hurtful to the country. 3. A large proportion of mankind being barred from the marriage state, for reasons indicated above and others, claim the right of a natural life, and become the strongest support of prostitution.

The *Medical Record*, in an editorial on the above subject, considers the increased birth-rate as a very small factor in producing distress in Germany, and refers to the expenses of keeping an army of half a million. As to the moral, it says: "The medical profession has, however, always been unanimous in discountenancing the tendency to seek irregular means for limiting the number of the family. * * * * The view that a low per cent. of births is a political necessity does not hold good in this country at any rate."

---

HIGH MORTALITY IN NEW YORK—THE CAUSES OF IT.—The city has never been in such an unsanitary condition before for such a long period, and has never been afflicted with such a relative mortality. * * * * Although the condition of our streets is, doubtless, the main cause of the malignity of disease and the high rate of mortality, it is not the only one. The drinking-water has been in a bad condition, and has been insufficient in supply. In many parts of the city, for months at a time, the pressure has been so low that water could not be obtained above the first stories of dwelling houses. As a consequence traps became dry, water closets were not flushed, and sewer-gas entered freely into the houses.

Again, the condition of the sewers must not be lost sight of. Aside from being improperly constructed, they have been compelled to find egress for the washings from the miles of gutters loaded with the rotten refuse of the tenement districts. Of course there is ventilation for the sewers through the houses by means of roof pipes, but it is a question whether the authorities will not now be compelled to get rid of the surplus of noxious gases in some other way.

And still again, it seems proper to take into account the influence of the nuisances of Hunter's Point, of the abattoirs on the east side, of the unsanitary condition of our public schools, and of the crowded state of our tenement districts. All these are factors of greater or less influence upon the mortality of our city, and should be duly estimated in the advocacy of reformatory measures. The streets must be cleaned, but other important matters must also receive attention.—*Medical Record.*

An outbreak of typhus fever has appeared in New York. On March 26th, there were thirty-four cases suffering from the disease in the Riverside Hospital. They were taken from their homes, lodging-houses, and from the German, Charity, Bellevue, St. Francis, and U. S. Marine Hospitals. All, except one from the latter hospital, have been traced to the Shiloh lodging-house, a cheap resort for tramps, which often accommodates five hundred persons nightly. Small-pox has been discovered in the same place, which is now under the care of the sanitary officials.—*Ibid.*

---

SCARCITY OF DISSECTING MATERIAL.—In one of the metropolitan schools of London but one subject had been received for dissection up to December, while the number of students awaiting material exceeded ninety. The object of the recent "Anatomy Act," was to increase facilities for studying anatomy, but it has signally failed. This state of affairs tallies with our own. Anatomy Acts here, stimulated by an indignant public, are paralyzing good medical training. Officers of public institutions, says the act, *shall* give up unclaimed bodies. There is no penalty incident to violating the act, and thus those officers openly avoid the true intent of the law. Dissection is a *sine qua non.* This fact is indisputable, axiomatic. The State must permit easy access of material. Officers of public institutions, where unclaimed bodies may lie, must be compelled to *notify* medical colleges, and suffer the peaceable removal of cadavers. Such action could not offend public morals, and must increase the facilities for medical teaching.

In Maine, a medical degree cannot be obtained without the doctor violating law; for he must dissect to fulfill the conditions of the degree, and a cadaver cannot legally be secured. Such absurdities are humiliating. The recent Ohio legislation has not been much better; but we hope the present legislature will take some sensible action in this very important matter.—*The Ohio Medical Recorder.*

---

CREMATION.—"At present Germany and Italy are the only European countries where incineration of the dead is really practiced. It is far more popular in the latter country than in the former, and Milan is now the chief cremating center. During the past four years over seventy bodies have been burned in the furnace in that city. There have been ten or fifteen cremations at Lodi, and a larger number at Gotha. The number is greater, however, each succeeding year, and there is every sign of a growing popularity of the practice. In England the sentiment of many of the very best men is either in its favor or not against it. The graveyards are a bane to England, and in some places another mode of disposing of the dead is almost a necessity. The Earl of Beaconsfield, the Bishop of Manchester, and others have spoken loudly upon the danger to health of the English churchyards. Since last summer quite a strong feeling in favor of cremation has shown itself among medical men. The total cost of a cremation at Milan is nearly ten dollars. To this must be added the cost of bringing the body to the crematorium.

"We have, before this, expressed our opinion in regard to the practice of cremation. From a sanitary point of view it is to be highly commended, and is, indeed, in some places, particularly in the crowded cities of the Old World, almost a necessity. The objections to it are made on æsthetic, on economic, and on medico-legal grounds. Regarding the first, none have successfully refuted the argument that it is only prejudice which makes the practice seem revolting or irreligious. It is claimed that cremation may destroy evidence in criminal cases. And it is certain that there will have to be special legal regulations of the practice. It is probable that after all cremation will succeed or fail, according as it does or does not afford a means of disposing of the dead more cheaply and decently than by burial. If cremations are expensive, and crematories offensive, they will not become popular, even though graveyards are unwholesome. The principles of economic science live after us. As far as experience goes now, it seems likely that cremations in large cities can be done cheaply. And here the sanitary value of the practice is most apparent. We shall watch with interest the work and progress of the New York Cremation Society."—*Medical Record.*

---

BELLEVUE HOSPITAL MEDICAL COLLEGE.—The faculty of this well-known college has returned to the two-years course of study. Only fifty new students were registered since last fall. This is not so good a result as Harvard's, quoted in this article. Perhaps we might consider the Medical Colleges as affording only preliminary training. The majority of practitioners attend post-graduate courses, study in hospitals, or abroad, or practice a few years with their preceptors, and very few pretend to refer their success in practice to the few data committed to memory during two medical courses. The advantages of lectures are always limited, and it is a question if more repetition would be followed by better results.

---

SMALL-POX HOSPITALS IN LONDON.—This caused some agitation in the London press and the House of Lords a short time ago. Vaccination in the metropolis has not been received with more favor than in Chicago, although the belief in its efficacy is deeply rooted in the medical profession of London. It is even asserted by the *British Medical Journal* that, as statistics regarding the attendants and nurses of small-pox hospitals show, persons efficiently vaccinated and re-vaccinated may live with impunity in the company of the variolous. But it seems that the nonacceptance of such views by the people at large is the cause that small-pox remains endemic and necessitates special hospitals, These are not without danger in a city like London, but it is acknowledged that ambulances are a more common source of risk, and should be so constructed as to be readily recognized.

"So long as vaccination remains in its present unsatisfactory condition, small-pox hospitals will be necessary; and we are of opinion that these hospitals may be so conducted as to be harmless to the neighborhoods in which they may be placed.—*The British Medical Journal, March* 12.

---

PROPOSED ROYAL SOCIETY OF MEDICINE IN ENGLAND.—The important question which Mr. Erichsen revived in the interesting address with which he concluded his prosperous and dignified presidency of the Royal Medical and Chirurgical Society, is one on which, we believe, a large preponderance of opinion will be found to support the conclusions to which his experience has conducted him. The advantages of the scheme are so palpable, that it might hardly seem necessary to set them forth. The great object of those who urge the amalgamation of the Royal Medical and Chirurgical, Pathological, and Clinical, and others, into a single society, is to prevent the waste of material and the overlapping which is so strongly felt under the present system; to render the new institution grander and more commensurate with the dignity and the increasing public importance of the science of medicine; more ready and more fit to assist the government and the public, on occasions of emergency, with counsel and assistance such as can hardly be obtained at present even from the colleges, and certainly from no other institution. Along with this it is believed that such an amalgamation will place the new Royal Society of Medicine on a firmer financial basis, and render its continuance more secure than can be said to be the case with the present societies—even the oldest and most dignified of them. The scheme was so near success in 1870 that it is almost difficult now to see why it was dropped. If it be heartily advocated and properly worked out now, we do not see why it should not be soon realized."—*Ibid.*

SWEATING DISEASE.—In July, 1880, an epidemic of sweating disease (*suette miliaire*) of which we know very little in this country, carried away 142 victims among 1,000 cases attacked, in a population of 20,000 at Oleron. This is an island off the coast of France in Biscay Bay. The same disease raged 31 years ago in three departments, and was scientifically investigated. It has been endemic since in various localities. It is an infectious disease, but it is doubtful if it be caught directly from one person by another ; it is more probable that it is communicated in the manner of Asiatic cholera. In 15 days the whole island had been invaded. Symptoms: An abrupt start, rapid evolution, profuse perspiration, a peculiar eruption, pain in the epigastrium, distress in breathing, often giving rise to a sense of suffocation, constipation and loss of sleep, fever and delirium. Death may supervene in 12 hours. Six hours after death the cadaver exhales a horrible stench, and a fetid blood sometimes runs out of the nostrils and mouth. This seems the origin of the contagium ; and as in Oleron the coffins are carried to the church-yards on men's shoulders, this might also partly explain the rapid spread of the epidemic. The temperature has ranged from 40° to 43° C., in grave cases (104° to 107° F.). Treatment: Ipecacuanha in scruple doses, 1 gr. 5, once in 24 hours, seemed very useful. Quinine is said to have failed in reducing the temperature. Cold packs were efficient; they encourage the eruption.—*Communication of Dr. Jules Rochard to the Académie, at its séance of March* 1, 1881.

---

BACTERIA AND FRENCH ENTHUSIASM.—The JOURNAL AND EXAMINER has given to its numerous subscribers, in November and February, faithful translations of some valuable experiments with bacteria in France. The subject has now created so much enthusiasm in that country, that we could not form an idea of it here, were it not for the injury done to American trade by the edict prohibiting the importation of American pork in France. We know that the edict followed the discovery of the *trichina spiralis* in some hams imported from America, although no epidemic, nor even individual deaths had been recorded. Some members of the Académie de Médecine have forgotten their dignity and quarreled over the experiments; and discoveries have been announced and echoed from the boundaries of the country before they had even been made.

However, there is something worthy the admiration of the scientific world in the fact that in the laboratories of the Parisian Normal School such mortal viruses as those of hydrophobia, of anthrax, and of septicæmia are daily manipulated by experimenters. The capital invested in these researches also deserves some attention. The state had to become interested in them to some degree before it would put at the disposition of experimenters the flocks of sheep of the ex-imperial farm at Vincennes, and those of the school of Alford. Some investigators were sent to Auvergne, Beauce, and even to Algeria by the State, and military veterinary surgeons were enjoined to assist them. The opposition so far has been vigorous enough to test, and thereby render more brilliant, the results which amount to this:

M. Semmer, of Dorpat, experimenting with due regard to the conditions advocated by Pasteur, has succeeded in vaccinating rabbits for septicæmia. M. Toussaint vaccinated sheep successfully for anthrax, and obtained the wonderful result of an hereditary immunity from that disease in the young. A short time ago, two veterinary professors of Lyons having vaccinated successfully for a form of anthrax in a locality where the disease is endemic, a cattle insurance company had 250 cattle of the bovine species vaccinated with the vibriones of the anthrax virus. Although these facts are not numerous yet, and involved in some obscurity, although they may not be so practical as they appear to such men as Pasteur and Toussaint, yet they are certainly interesting, and it is a pity that, with the vast resources afforded by the government, state boards of health, and especially by private fortunes here, they are so little investigated.

